# A genome-wide landscape of mRNAs, lncRNAs, and circRNAs during subcutaneous adipogenesis in pigs

**DOI:** 10.1186/s40104-018-0292-7

**Published:** 2018-11-01

**Authors:** Xin Liu, Kaiqing Liu, Baosen Shan, Shengjuan Wei, Dongfeng Li, Haiyin Han, Wei Wei, Jie Chen, Honglin Liu, Lifan Zhang

**Affiliations:** 1 College of Animal Science and Technology, Nanjing Agricultural University, Nanjing, 210095 China; 2 College of Life Sciences and Food Engineering, Hebei University of Engineering, Handan, 056038 China

**Keywords:** CircRNA, LncRNA, mRNA, Pig, Preadipocyte differentiation, RNA-Seq

## Abstract

**Background:**

Preadipocyte differentiation plays a critical role in subcutaneous fat deposition in pigs. However, the roles of different RNAs, such as messenger RNAs (mRNAs), long non-coding RNAs (lncRNAs), and circular RNAs (circRNAs) in the differentiation process of subcutaneous preadipocytes, are still largely unclear. In the present study, a transcriptome analysis, including the analysis of mRNAs, lncRNAs, and circRNAs, during different differentiation stages, namely, day 0 (D0), day 2 (D2), day 4 (D4), and day 8 (D8), of subcutaneous preadipocytes from Chinese Erhualian pigs was performed.

**Results:**

A total of 1554, 470, 1344, 1777, and 676 differentially expressed (DE) mRNAs, 112, 58, 95, 136, and 93 DE lncRNAs, and 902, 787, 710, 932, and 850 DE circRNAs were identified between D2 and D0, between D4 and D2, between D8 and D4, between D4 and D0, and between D8 and D0, respectively. Furthermore, functional enrichment analysis showed that the common DE mRNAs during the entire differentiation process were mainly involved in lipid metabolic and cell differentiation processes. Additionally, co-expression network analysis identified the potential lncRNAs related to adipogenesis, e.g., MSTRG.131380 and MSTRG.62128.

**Conclusions:**

Our study provides new insights of the expression changes of RNAs during adipogenic differentiation, which might contribute to the phenotype of subcutaneous adipogenesis. These results greatly improve our understanding of the molecular mechanisms regulating subcutaneous fat deposition in pigs.

**Electronic supplementary material:**

The online version of this article (10.1186/s40104-018-0292-7) contains supplementary material, which is available to authorized users.

## Background

Being a crucial part of body energy metabolism and the endocrine system, subcutaneous fat plays an important role in the growth and meat quality of pigs. On one hand, excessive subcutaneous fat deposition in pigs, especially in obese pig breeds, greatly decreases the growth performance and production efficiency, which results in high costs of feeding and production. On the other hand, a sufficient layer of subcutaneous fat is necessary to obtain high quality processed products such as dry-cured hams [1, 2]. Additionally, the thickness of subcutaneous fat has been reported to positively correlate with intramuscular fat (IMF) in the longissimus dorsi and gluteus medius muscles [3]. Therefore, understanding the mechanism of subcutaneous fat formation would be greatly beneficial to improve the production efficiency and meat quality. Because the increase in the size of porcine adipocytes is closely related with adipose tissue expansion, controlling subcutaneous preadipocyte differentiation could be considered a good strategy for regulating subcutaneous fat management.

In recent years, two main types of noncoding RNAs (ncRNAs), including long non-coding RNAs (lncRNAs) and circular RNAs (circRNAs), have been regarded as key regulators, because they play a vital role in regulating gene expressions at the transcriptional and post-transcriptional levels [4, 5]. LncRNAs are one class of ncRNAs that are longer than 200 nucleotides in length, but contain no open reading frames [6]; they have been revealed to affect preadipocyte differentiation by regulating adipogenic-related key genes such as *PPARG* and *CEBPA* in humans and mice [7–9], demonstrating that lncRNAs might have an essential role in adipogenesis. CircRNAs are a unique class of ncRNAs with a covalently closed continuous loop without 5′ caps and 3′ tails; they have been shown to be widely expressed in animal tissues and cells [10]. More interestingly, certain circRNAs have tissue-specific and stage-specific expression patterns [11], indicating that circRNAs would be a specific type of regulator in cell or tissue development processes. Additionally, accumulating evidences indicate that circRNAs have an important role in mammalian cell differentiation [12, 13]. However, thus far, the functions of lncRNAs and circRNAs in porcine subcutaneous preadipocyte differentiation are still largely unknown.

Previously, several studies discovered the messenger RNA (mRNA) or microRNA (miRNA) expression profiles of subcutaneous tissues between high and low backfat pigs [14, 15]. Furthermore, the changes of mRNAs in subcutaneous preadipocytes during adipogenic differentiation were studied in pigs and mice [16, 17]. Moreover, lncRNAs related to castration-induced subcutaneous fat changes were identified in Huainan male pigs [18]. However, the expression profiling of RNAs during porcine subcutaneous preadipocyte differentiation has not yet been well studied. For example, the mRNA transcriptome analysis of porcine subcutaneous preadipocytes during their differentiation is only performed at the early and middle stages of differentiation in Large White pigs [16]. Furthermore, little is known about the expression characteristics of lncRNAs and circRNAs during adipogenic differentiation. Accordingly, it is necessary to further analyze the expression patterns of RNAs, including coding and noncoding RNAs, during porcine subcutaneous preadipocyte differentiation. Additionally, as a typical indigenous pig breed with plenty of subcutaneous fat, the Chinese Erhualian pig is a good model for studying subcutaneous fat formation. As such, the expression characters of mRNAs, lncRNAs, and circRNAs during different differentiation stages (day 0 (D0), day 2 (D2), day 4 (D4), and day 8 (D8)) of subcutaneous preadipocytes in Erhualian pigs were investigated using RNA sequencing (RNA-Seq) technology. Our results demonstrate the genome-wide changes of molecular events during adipogenic differentiation, thus giving us newer insights regarding subcutaneous fat management of pigs.

## Methods

### Animals

Three five-day-old Chinese Erhualian piglets were purchased from Changzhou Erhualian Pig Production Cooperation (Changzhou, Jiangsu, China). The experimental procedures were approved by the Institutional Animal Care and Use Committee of Nanjing Agricultural University.

### Preadipocyte culture and differentiation

Newly isolated subcutaneous adipose tissue from each piglet was washed thrice with phosphate-buffered saline (PBS). Then, the tissue was minced and digested with 1 mg/mL collagenase type I (Invitrogen, Carlsbad, CA, USA) at 37 °C for 60 min, followed by filtration through a 200 μm nylon mesh for removing the undigested fractions. The filtrated solution was centrifuged at 1,000 r/min for 10 min to collect the preadipocytes, and then, the cells were cultured in Dulbecco’s modified Eagle’s medium/Ham’s F-12 (DMEM-F12) growth medium containing 10% fetal bovine serum (FBS) and 1% penicillin-streptomycin at 37 °C with an atmosphere of 5% CO_2_. After the preadipocytes reached confluence (D0), the DMI hormone cocktail (1 μmol/L dexamethasone, 0.5 mmol/L 3-isobutyl-1-methylxanthine, and 5 μg/mL insulin) was added to the growth medium to induce the cell differentiation for 2 d. Next, the cells were subjected to maintenance medium (growth medium supplemented with 5 μg/mL insulin) for an additional 2 d. After that, the growth medium was changed every alternate day until adipocyte maturation.

### Oil Red O staining and triglyceride assay

After removing the culture medium, the adipocytes were washed thrice with PBS and fixed in 10% formaldehyde for 15 min. Next, the cells were washed thrice with PBS, and then stained with Oil Red O for 20 min. Finally, the cells were washed thrice with PBS and photographed using an inverted microscope (Leica, Wetzlar, Germany). The absorbance values of Oil Red O-stained cells were measured at the wavelength of 510 nm to quantify the lipid accumulation. Meanwhile, triglyceride contents were determined using a commercial triglyceride assay kit (Applygen, Beijing, China), according to the manufacturer’s protocol.

### RNA extraction, library preparation, and sequencing

Total RNA was isolated at each time point (D0, D2, D4, and D8) using the Trizol reagent (Invitrogen, Carlsbad, CA, USA). The qualities and quantities of the RNA were measured using Bioanalyzer 2100 (Agilent Technologies, Santa Clare, CA, USA) and 1% agarose gel electrophoresis, which showed that the RNA integrity number (RIN) values of all samples were 10. Ribosomal RNA from each sample was removed using the Ribo-Zero™ GoldKits (Epicentre, Madison, WI, USA). Equal amounts of total RNA of the same stage of differentiation from three Erhualian piglets were pooled into one sample. Then, cDNA libraries were prepared using a NEB Next Ultra Directional RNA LibraryPrep Kit (NEB, Ispawich, MA, USA), according the manufacturer’s instructions and sequenced using the Illumina HiSeq X Ten system (Illumina, San Diego, CA, USA).

### Identification of lncRNA and circRNA

Low-quality and adaptor-polluted reads were firstly removed from the raw data. The clean reads from each sample were aligned onto the pig reference genome (*Sus scrofa* 11.1) using the HiSAT2 v2.0.5 program [19]. In addition, because the sequences of circRNA cannot be directly aligned to the reference genome, the slicing alignment was mapped to the genome for obtaining the circRNA using the Burrows-Wheeler Aligner-maximal exact match (BWA-MEM) algorithm [20]. The candidate lncRNAs were identified using the following criteria: 1) transcripts were filtered by removing those shorter than 200 nt, those with less than two exons, and those with a read coverage less than five in all samples, to avoid unreliable transcripts or those with inconsistent lengths, and sequences consisting of the known mRNAs and other non-coding RNAs (ribosomal RNA (rRNA), transfer RNA (tRNA), small nucleolar RNA (snoRNA), and small nuclear RNA (snRNA)); 2) putative lncRNAs for their protein-coding ability were determined using four approaches, including coding-non-coding index (CNCI), coding potential calculator (CPC), protein folding domain database (PFAM), and coding potential assessing tool (CPAT). Finally, the remaining transcripts were defined as novel lncRNAs. Furthermore, the candidate circRNAs were recognized using the CIRI (circRNA identifier) algorithm [21]. In brief, paired chiastic clipping, paired-end mapping, and GT-AG splicing signals were discovered via scanning the above obtained slicing alignments. Next, the alignment files were scanned again using a dynamic programming algorithm for detecting additional junction reads and eliminating false positive circRNA candidates. The final circRNAs were obtained by retaining sequences with ≥2 junction reads.

### Analysis of differentially expressed (DE) genes

The fragments per kilobase of transcript per million reads mapped (FPKM) value was used to estimate the expression levels of mRNAs and lncRNAs, while the spliced reads per billion mapping (SRPBM) value was utilized to determine the amount of circRNAs [22]. Genes with an FPKM or SRPBM value of < 1 in no less than 50% of samples were defined as unreliably expressed genes, while those with an FPKM or SRPBM value of ≥1 in more than 50% of samples were considered as reliably expressed genes. Differentially expressed (DE) genes including mRNAs, lncRNAs, and circRNAs were analyzed using DEGseq v1.18.0 [23], which defined DE genes as reliably expressed genes with |log_2__ratio| ≥ 1 and false discovery rate (FDR) < 0.05 between any two groups. Meanwhile, genes differentially expressed in three comparisons (D2 versus D0, D4 versus D0, and D8 versus D0) were defined as common DE genes.

### Gene ontology (GO) analysis

The function of DE lncRNAs was predicted by the GO analysis of their *cis*- and *trans*-target mRNAs, which were screened based on their genomic positional relation 50 kilobase pairs (kb) upstream and 50 kb downstream, for *cis*-target mRNAs and based on the Pearson correlation coefficient of lncRNA-RNA pairs being ≥0.9, for *trans*-target mRNAs. The function of DE circRNAs was revealed via GO analysis of their parental genes. In brief, all genes were firstly mapped to GO terms using the Gene Ontology database (http://www.geneontology.org), and then, the functional enrichment analysis was performed using Omicshare tools (www.omicshare.com/tools). Terms with *P*-values less than 0.01 and more than 3 matching genes were identified as significant or enriched terms.

### Co-expression network construction

The co-expression network of common DE lncRNAs with their *cis*- and *trans*-target common DE mRNAs was constructed using the Cytoscape software (v3.2.1) to explore the function of key lncRNAs [24].

### Quantitative real-time RT-PCR

Primers used in quantitative real-time RT-PCR (qRT-PCR) were listed in Additional file 1. Three RNAs samples per differentiation stage were reverse transcribed using a PrimeScript™ Master Mix (Takara, Dalian, China), according to the manufacturer’s instructions. Next, qRT-PCR was performed using SYBR Premix Ex Taq™ (Takara, Dalian, China) on the StepOnePlus™ Real-time PCR System (Applied Biosystems, Foster City, CA, USA). The reaction conditions were as follows: denaturation for 30 s at 95 °C, followed by 40 cycles of 95 °C for 5 s and 60 °C for 30 s. Meanwhile, *RPLP0* (ribosomal protein lateral stalk subunit P0), *PPIA* (peptidylprolyl isomerase A), and *HPRT1* (hypoxanthine phosphoribosyltransferase 1) were used as the normalization controls, and the experiments were performed in triplicate. The 2^-ΔΔCT^ method was used to calculate the relative gene expression levels.

### Statistical analysis

Statistical analysis of the data from triglyceride and qRT-PCR assay was performed using the SPSS software (version 20.0). Statistical comparison among the groups was analyzed using one-way analysis of variance (ANOVA), followed by Tukey’s multiple comparison test. *P*-values less than 0.05 were considered significant, while *P*-values less than 0.01 or 0.001 were considered highly significant.

### Data accessibility

The sequencing data have been submitted to the Gene Expression Omnibus (GEO) database (accession number GSE114583).

## Results

### Phenotypic changes during preadipocyte differentiation

**Fig. 1:**
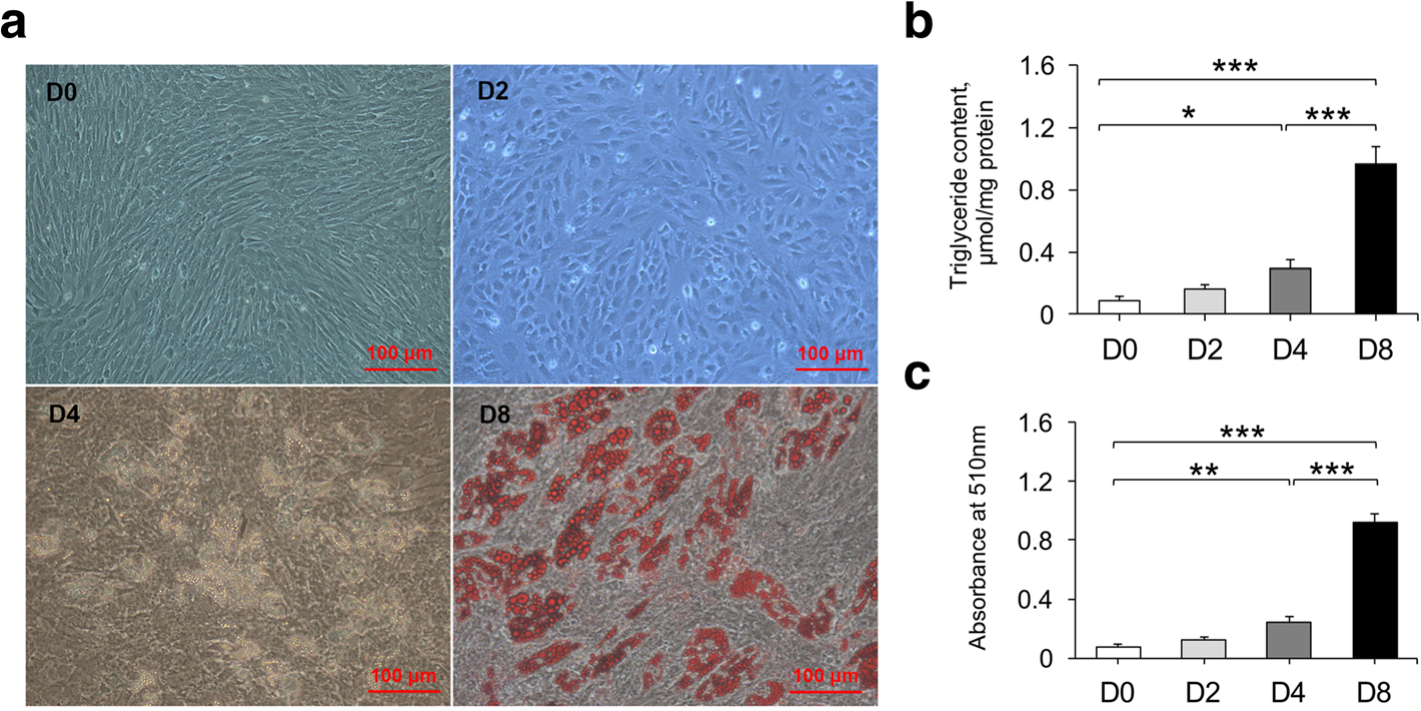
In vitro adipocyte differentiation. Adipocytes were obtained from the subcutaneous adipose tissue of three five-day-old Chinese Erhualian pigs and collected at four differentiation stages: D0, D2, D4, and D8. (**a**) Enlarged adipocyte photos during the differentiation (D0, D2, D4, and D8; D8 with Oil Red O staining) (200×). (**b**) Triglyceride contents at the different stages of differentiation were measured using a triglycerol assay kit. (**c**) Absorbance values of destained Oil Red O extracted from the cells at 510 nm. Data are represented as mean ± SD, *n* = 3 per group. * *P* < 0.05; ** *P* < 0.01; *** *P* < 0.001

Compared to the cell shapes in the initial phase (D0), the preadipocytes gradually turned from fibrous into a spherical shape at D2. Subsequently, lipid droplets were visibly observed at D4 and gradually increased until D8 (Fig. 1a). In accordance with the results of the adipocyte shapes, the triglyceride levels progressively accumulated, accompanying the increase of the differentiation process (Fig. 1b-c). These data indicate that the subcutaneous preadipocyte differentiation process is normal and can be further analyzed.

### Characters of RNA-seq

After quality control, a total of 155981654, 137762268, 168562368, and 119115476 clean reads with greater than 94.80% of Q30 were obtained in D0, D2, D4, and D8, respectively (Table 1). Among them, a total of 97.49%, 97.60%, 97.66%, and 97.88% reads from D0, D2, D4, and D8, respectively, were mapped to the pig reference genome (*Sus scrofa* 11.1) (Table 1). Additionally, BWA-MEM was used for sequence split comparison to accurately identify circRNAs; a mapping rate of more than 99.91% from each sample was discovered (Table 1).

**Table 1:** Basic data of sequencing in four stages of adipocyte differentiation

Terms	D0	D2	D4	D8
Raw reads number	189,475,072	165,990,894	201,125,216	139,876,842
Clean reads number	155,981,654	137,762,268	168,562,368	119,115,476
Clean reads rate, %	82.32	82.99	83.81	85.16
Clean Q30 bases rate, %	94.99	94.80	94.81	95.35
Mapped reads	152,068,735	134,451,565	164,621,381	116,588,244
	(155,834,560)	(137,634,429)	(168,443,435)	(119,037,090)
Mapping rate, %	97.49	97.60	97.66	97.88
	(99.91)	(99.91)	(99.93)	(99.93)

The values outside the brackets represent the reads and proportion that were compared to those in the pig reference genome (*Sus scrofa* 11.1) using the HiSAT2 program, while the values inside the brackets represent the reads and proportion that were compared to those in the pig reference genome (*Sus scrofa* 11.1) using the BWA-MEM algorithm

### Gene expression profiles during preadipocyte differentiation

**Fig. 2:**
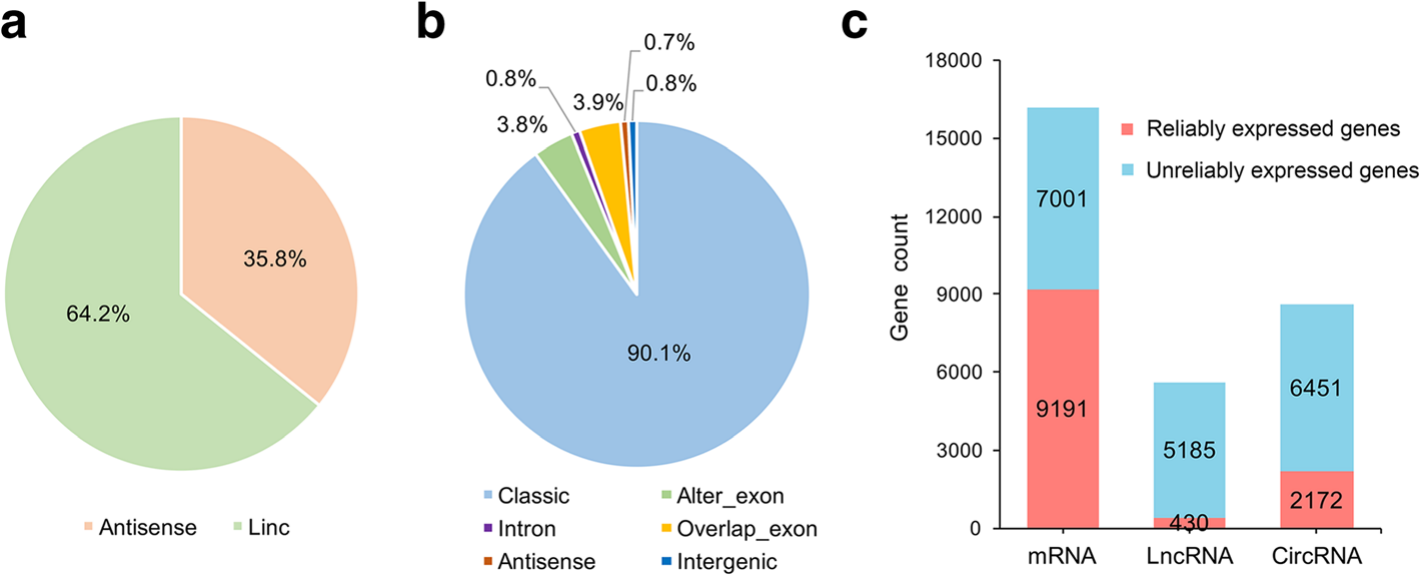
Gene expression characterization. (**a**) The type and proportion of novel long non-coding RNAs (lncRNAs). (**b**) The type and proportion of circular RNAs (circRNAs). (**c**) The number of reliably expressed genes (red, FPKM or SRPBM ≥1 in more than 50% of the samples) and unreliably expressed genes (blue, FPKM or SRPBM < 1 in no less than 50% of the samples)

**Fig. 3:**
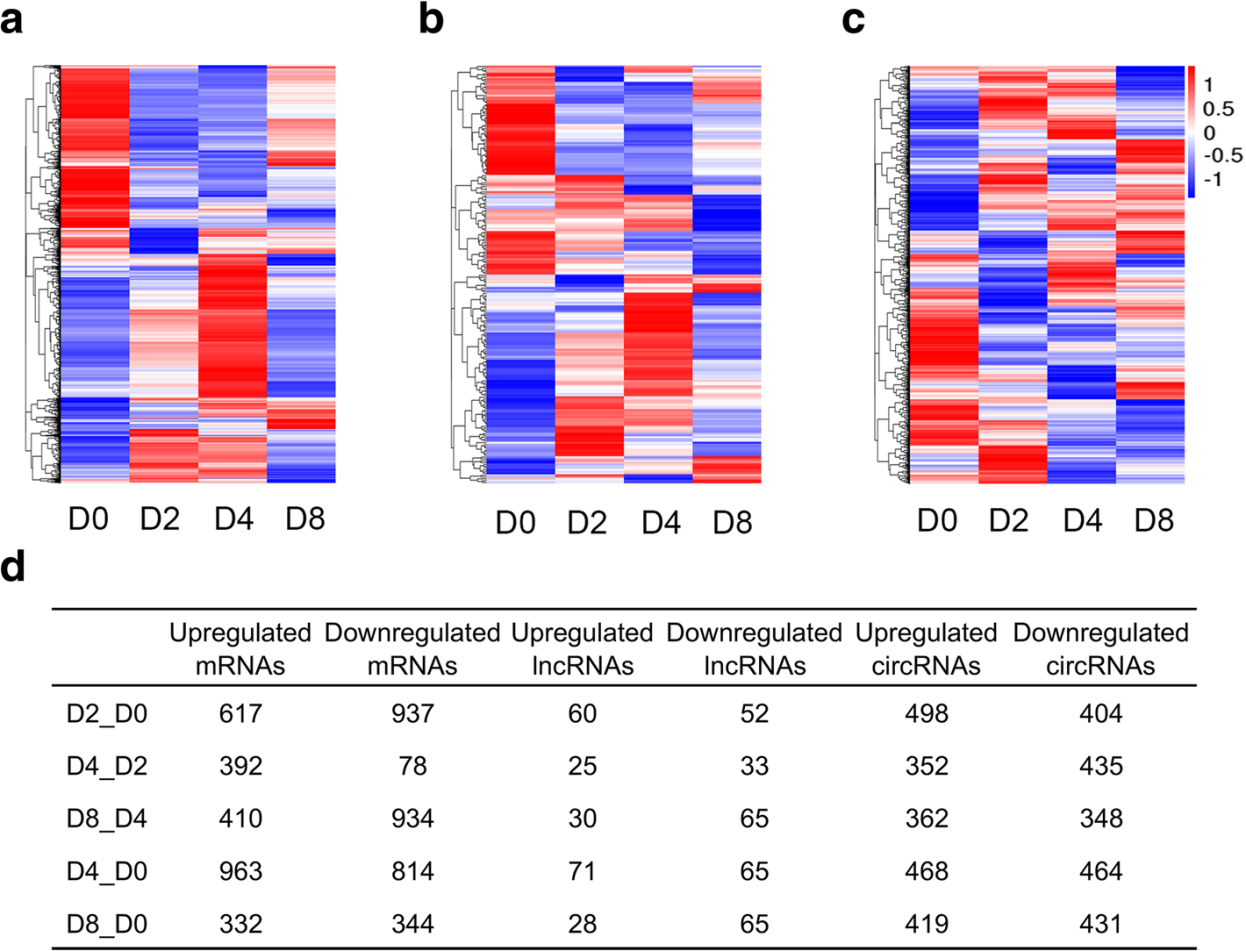
Differentially expressed (DE) genes and their expression modes. Heat map of all DE mRNAs (**a**), lncRNAs (**b**), and circRNAs (**c**) among the five compared groups. (**d**) The number of DE mRNAs, lncRNAs, and circRNAs in the D2 vs D0, D4 vs D2, D8 vs D4, D4 vs D0, and D8 vs D0 groups

A total of 16192 mRNAs, 5615 lncRNAs, and 8623 circRNAs were obtained from four stages (D0, D2, D4, and D8) of differentiation. LincRNAs and classic circRNAs accounted for the maximum proportion of novel lncRNAs and circRNAs, respectively (Fig. 2a-b). Based on an FPKM or SRPBM value of ≥1 in more than 2 groups, 9191 mRNAs, 430 lncRNAs (including 208 annotated lncRNAs and 222 novel lncRNAs), and 2172 circRNAs were identified as reliably expressed genes (Fig. 2c and Additional file 2). Furthermore, five comparison groups (D2 vs D0, D4 vs D2, D8 vs D4, D4 vs D0, and D8 vs D0) were set to discover the differentially expressed (DE) genes during the differentiation. A total of 2758 mRNAs, 230 lncRNAs, and 1760 circRNAs were defined as DE genes among these comparison groups (Fig. 3a-c). Of these, 1554, 470, 1344, 1777, and 676 DE mRNAs, 112, 58, 95, 136, and 93 DE lncRNAs, and 902, 787, 710, 932, and 850 DE circRNAs were identified between D2 and D0, between D4 and D2, between D8 and D4, between D4 and D0, and between D8 and D0, respectively (Fig. 3d and Additional files 3, 4, 5, 6 and 7).

### Gene ontology (GO) analysis of DE genes between D2 and D0

**Fig. 4:**
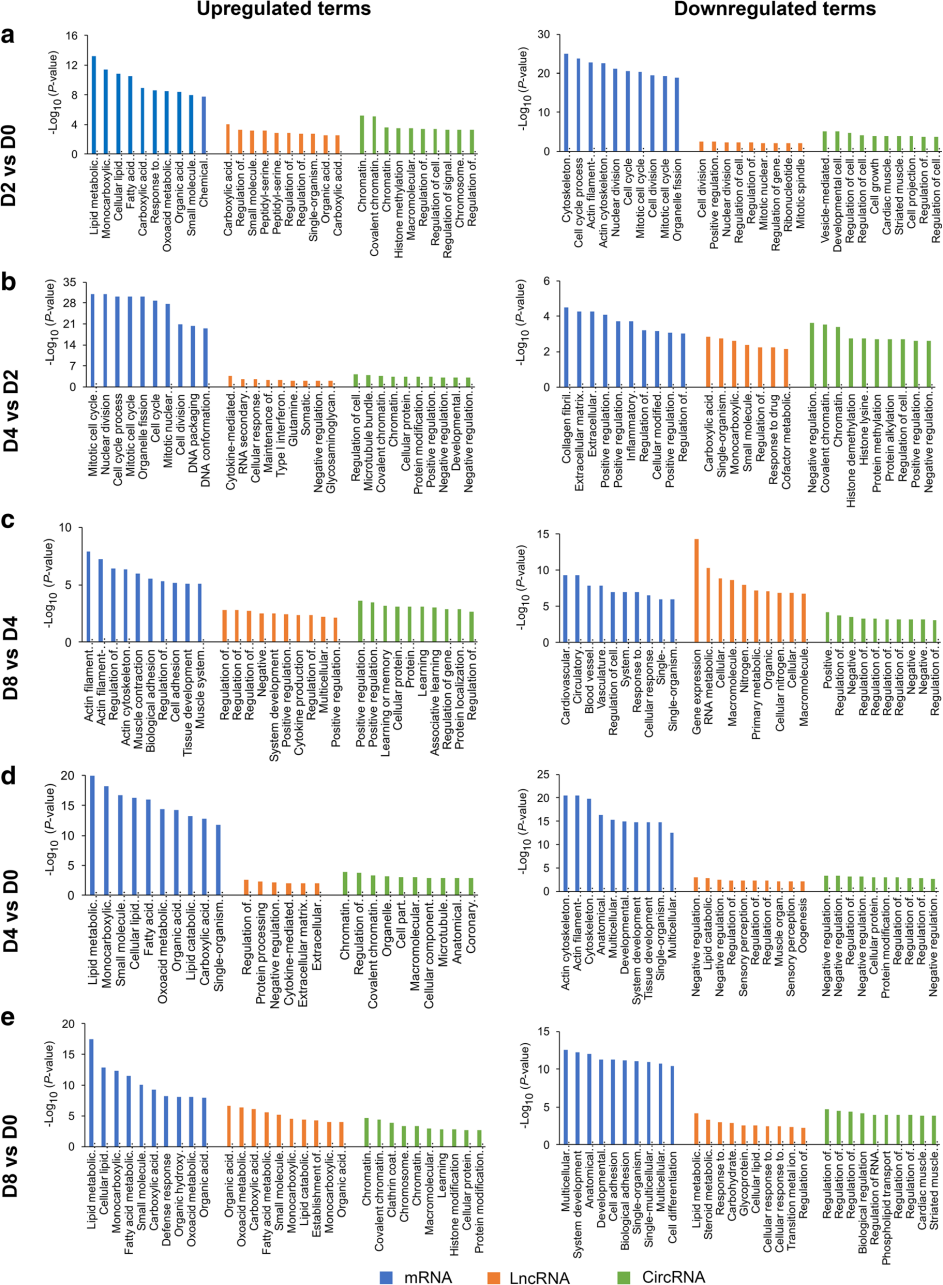
Gene ontology (GO) analysis. GO analysis of DE genes (including mRNAs, lncRNAs, and circRNAs) in the D2 vs D0, D4 vs D2, D8 vs D4, D4 vs D0, and D8 vs D0 groups. (**a**) GO analysis of DE genes in the D2 vs D0 group. (**b**) GO analysis of DE genes in the D4 vs D2 group. (**c**) GO analysis of DE genes in the D8 vs D4 group. (**d**) GO analysis of DE genes in the D4 vs D0 group. (**e**) GO analysis of DE genes in the D8 vs D0 group. The top 10 enriched GO terms ranked by *P*-values are shown

Between D2 and D0, significantly upregulated GO terms were mainly involved in: 1) lipid metabolism-related processes, response to oxygen-containing compound, and organic acid metabolic process for DE mRNAs, 2) carboxylic acid metabolic process, regulation of developmental growth, and small molecule catabolic process for DE lncRNAs, and 3) chromatin modification, histone methylation, and regulation of cell communication for DE circRNAs (Fig. 4a and Additional file 8). Additionally, significantly downregulated GO terms between D2 and D0 were mainly related to: 1) cytoskeleton organization and cell cycle-related processes for DE mRNAs, 2) positive regulation of developmental growth and cell cycle-related processes for DE lncRNAs, and 3) vesicle-mediated transport, developmental cell growth, and regulation of cell morphogenesis for DE circRNAs (Fig. 4a and Additional file 8).

### Gene ontology (GO) analysis of DE genes between D4 and D2

Between D4 and D2, significantly upregulated GO terms were mainly involved in: 1) cell cycle-related processes and DNA packaging for DE mRNAs, 2) cytokine-mediated signaling pathway, RNA secondary structure unwinding, and cellular response to type I interferon for DE lncRNAs, and 3) regulation of cell morphogenesis, microtubule bundle formation, and chromatin modification for DE circRNAs (Fig. 4b and Additional file 9). Additionally, significantly downregulated GO terms between D4 and D2 were mainly related to: 1) collagen fibril organization, extracellular matrix organization, and positive regulation of multicellular organismal process for DE mRNAs, 2) carboxylic acid metabolic process, single-organism catabolic process, and regulation of hormone levels for DE lncRNAs, and 3) chromatin modification, histone demethylation, and regulation of cell communication for DE circRNAs (Fig. 4b and Additional file 9).

### Gene ontology (GO) analysis of DE genes between D8 and D4

Between D8 and D4, significantly upregulated GO terms were mainly involved in: 1) actin cytoskeleton organization-related processes and biological adhesion for DE mRNAs, 2) regulation of protein processing, negative regulation of cell proliferation, and system development for DE lncRNAs, and 3) regulation of posttranscriptional gene silencing, protein modification process, and protein localization to Golgi apparatus for DE circRNAs (Fig. 4c and Additional file 10). Additionally, significantly downregulated GO terms between D8 and D4 were mainly related to: 1) regulation of cell proliferation, system development, and single-multicellular organism process for DE mRNAs, 2) gene expression, RNA metabolic process, and macromolecule metabolic process for DE lncRNAs, and 3) positive regulation of gene expression, regulation of nucleobase-containing compound metabolic process, and regulation of nitrogen compound metabolic process for DE circRNAs (Fig. 4c and Additional file 10).

### Gene ontology (GO) analysis of DE genes between D4 and D0

Between D4 and D0, significantly upregulated GO terms were mainly involved in: 1) lipid metabolism-related processes and monocarboxylic acid metabolic process for DE mRNAs, 2) regulation of hormone levels, protein processing, and cytokine-mediated signaling pathway for DE lncRNAs, and 3) chromatin modification, organelle organization, and cell part morphogenesis for DE circRNAs (Fig. 4d and Additional file 11). Furthmore, significantly downregulated GO terms were mainly enriched in: 1) cytoskeleton organization-related processes and multicellular organismal process for DE mRNAs, 2) negative regulation of growth, lipid catabolic process, and regulation of osteoclast differentiation for DE lncRNAs, and 3) negative regulation of macromolecule metabolic process, negative regulation of gene expression, and cellular protein modification process for DE circRNAs (Fig. 4d and Additional file 11).

### Gene ontology (GO) analysis of DE genes between D8 and D0

Between D8 and D0, significantly upregulated GO terms were mainly involved in: 1) lipid metabolism-related processes and small molecule metabolic process for DE mRNAs and lncRNAs, and 2) chromatin modification, clathrin coat assembly, and chromosome organization for DE circRNAs (Fig. 4e and Additional file 12). Furthmore, significantly downregulated GO terms were mainly related to: 1) multicellular organismal process, system development, and cell adhesion for DE mRNAs, 2) lipid metabolic process, steroid metabolic process, and carbohydrate derivative metabolic process for DE lncRNAs, and 3) regulation of cellular process, regulation of nucleobase-containing compound metabolic process, and regulation of biological process for DE circRNAs (Fig. 4e and Additional file 12).

### Function analysis of common DE genes during preadipocyte differentiation

**Fig. 5:**
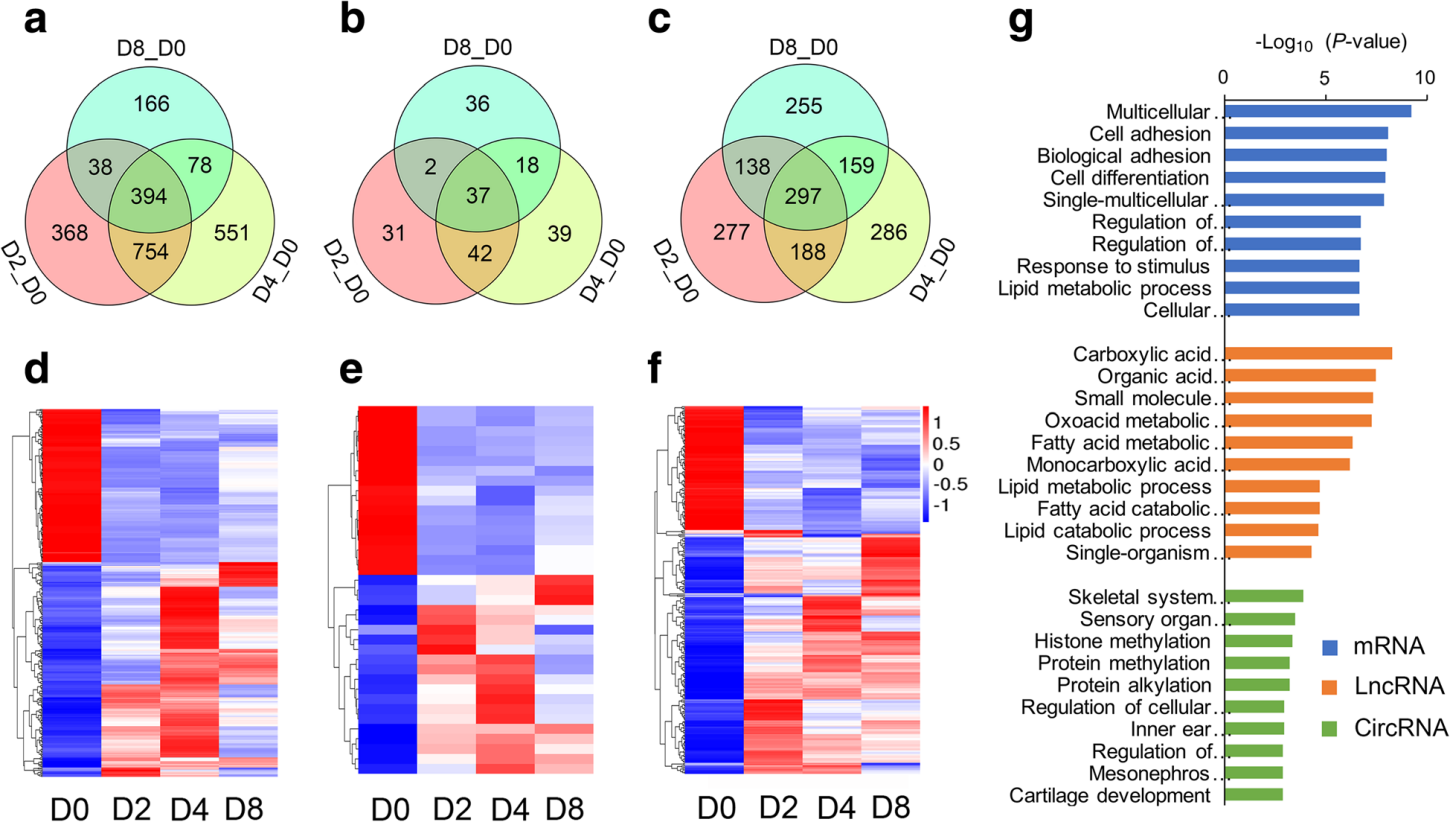
Expression analysis of common DE genes during the differentiation process. The number of common DE mRNAs (**a**), lncRNAs (**b**), and circRNAs (**c**). The expression levels of common DE mRNAs (**d**), lncRNAs (**e**), and circRNAs (**f**). (**g**) GO analysis of common DE genes. The top 10 enriched GO terms ranked by *P*-values are shown

Compared to the initial phase of differentiation (D0), 394 mRNAs, 37 lncRNAs, and 297 circRNAs were identified as common DE genes during the entire differentiation process (Fig. 5a-f and Additional file 13). GO analysis indicated that the terms were mainly enriched in: 1) cell adhesion, cell differentiation, and lipid metabolic process for DE mRNAs, 2) organic acid metabolic process, lipid metabolism-related processes, and single-organism process for DE lncRNAs, and 3) histone methylation, protein alkylation, and regulation of cellular response to growth factor stimulus for DE circRNAs (Fig. 5g and Additional file 14).

### Construction of the lncRNA-mRNA co-expression network

**Fig. 6:**
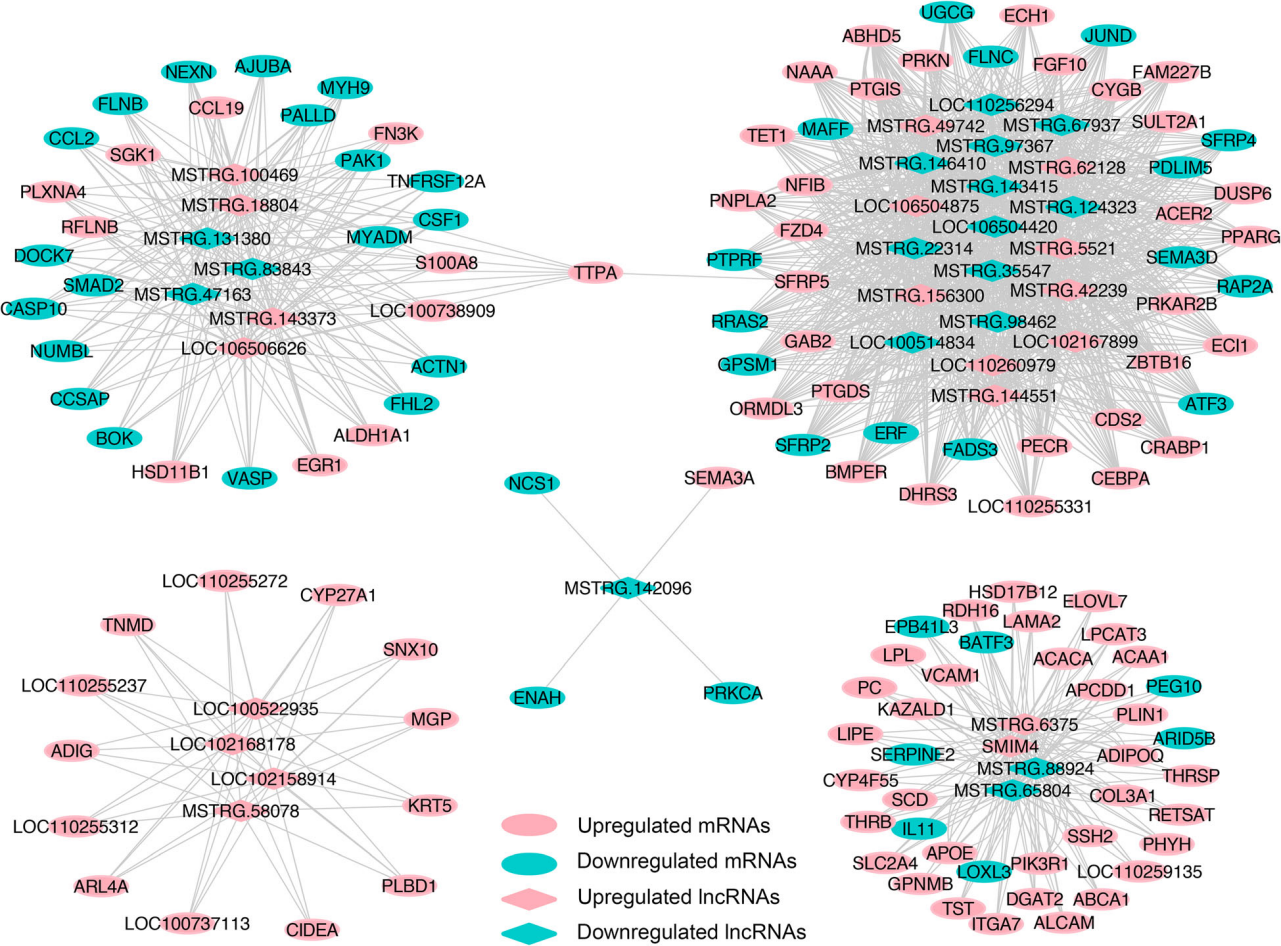
Co-expressed network construction. Co-expressed network of common DE lncRNAs and their targeted common DE mRNAs involved in lipid metabolic and cell differentiation processes. Pink and cyan ellipses represent upregulated and downregulated mRNAs, respectively, while pink and cyan diamonds represent upregulated and downregulated lncRNAs, respectively

To identify the key lncRNAs related to the regulation of lipid metabolic and cell differentiation processes, 132 common DE mRNAs associated with these two processes and 36 common DE lncRNAs targeting them were chosen to build the mRNA-lncRNA co-expression network. The results demonstrated that the co-expression network comprised 1328 connections and each lncRNA might correlate with multiple mRNAs (Fig. 6 and Additional file 15). More importantly, a total of 20 lncRNAs were found to be co-expressed with *PPARG*, a key adipocyte differentiation marker, while four lncRNAs including MSTRG.6375, *SMIM4*, MSTRG.88924, and MSTRG.65804 were shown to be co-expressed with other lipid metabolism-related markers such as *APOE*, *LIPE*, and *ADIPOQ* (Fig. 6 and Additional file 15), indicating that these lncRNAs might play an important role in regulating adipogenesis.

### Validation of DE genes by qRT-PCR

**Fig. 7:**
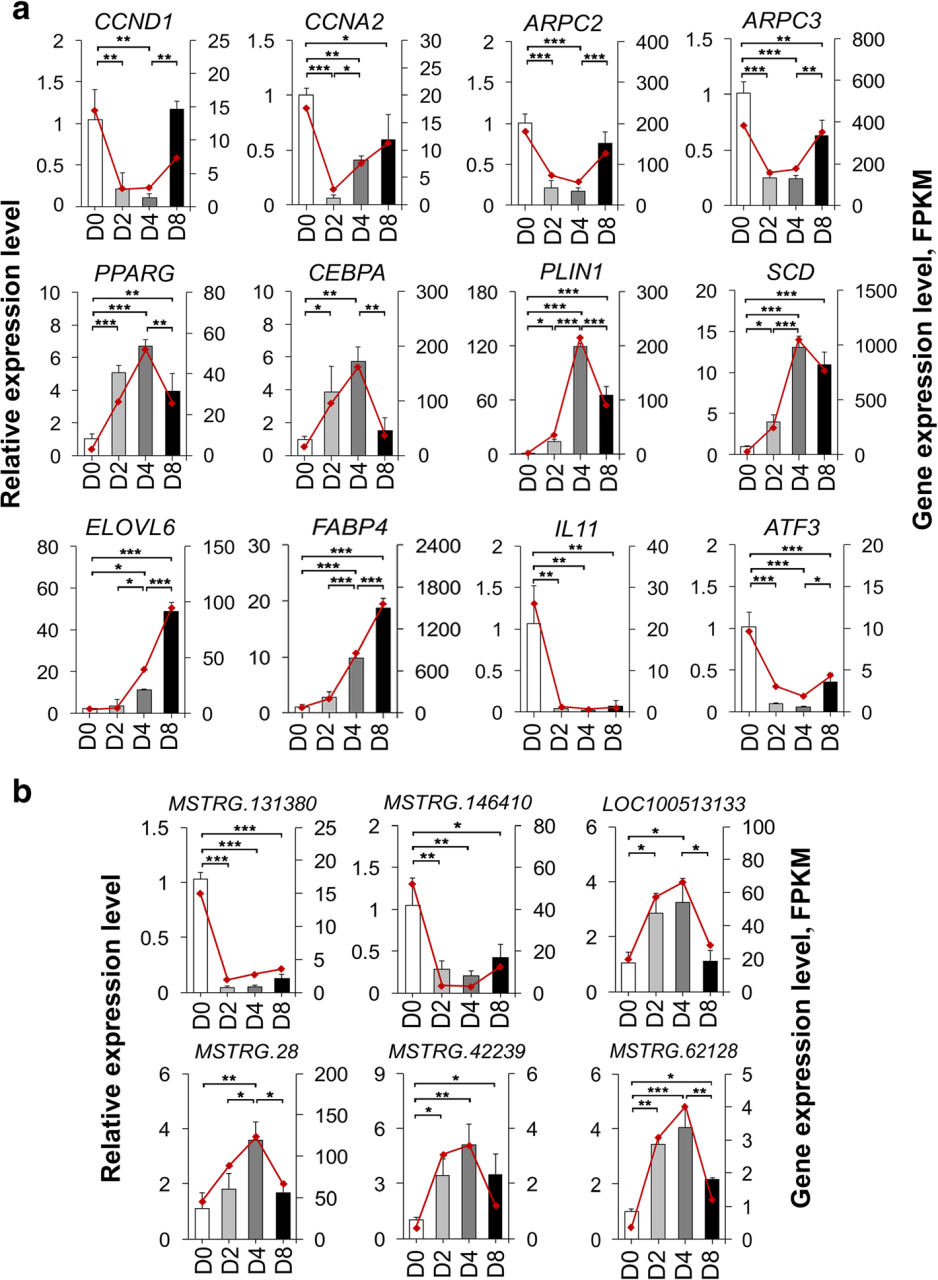
Validation of the expression of DE genes by qRT-PCR. qRT-PCR validation of the expression levels of twelve DE mRNAs associated with cell cycle, actin cytoskeleton, cell differentiation, and lipid metabolism process (**a**) and six randomly selected DE lncRNAs (**b**) in the four differentiation stages. Data from qRT-PCR are shown as column and *Y*-axis on the left, while the data from RNA-Seq are shown as line and *Y*-axis on the right. Data are represented as mean ± SD, *n* = 3 per group. * *P* < 0.05; ** *P* < 0.01; *** *P* < 0.001

A total of eighteen genes, including twelve mRNAs (*CCND1*, *CCNA2*, *ARPC2*, *ARPC3*, *PPARG*, *CEBPA*, *PLIN1*, *SCD*, *ELOVL6*, *FABP4*, *IL11*, and *ATF3*) related to cell cycle, actin cytoskeleton, cell differentiation, and lipid metabolism and six random lncRNAs (MSTRG.131380, MSTRG.146410, LOC100513133, MSTRG.28, MSTRG.42239, and MSTRG.62128), were selected for qRT-PCR verification. After comparison with the RNA-Seq data, similar expression trends for qRT-PCR were discovered, showing the strong consistency between qRT-PCR and RNA-Seq data (Fig. 7a-b).

## Discussion

As a key physiological process of normal body fat storage, preadipocyte differentiation provides a great opportunity for resolving the formation of fat deposition. In the present study, we observed that the shape of subcutaneous adipocytes changed from the shuttle-like form into the circlular form at the early stage of differentiation (D2), compared to the initial phase (D0) (Fig. 1a). Fat droplets were produced at D4, and then, clusters of large lipid droplets were formed at D8 (Fig. 1a). Consistent with the morphological changes of the adipocytes, triglyceride contents were observed to gradually increase, accompanying the increase in the differentiation time, strongly supporting the fact that preadipocyte differentiation is a complex process including both adipocyte growth and lipid deposits (Fig. 1b-c). Meanwhile, we identified more than 470 DE mRNAs, 58 DE lncRNAs, and 710 DE circRNAs among the different differentiation stages (Fig. 3d and Additional files 3, 4, 5, 6 and 7), which were involved in multiple biological processes including lipid metabolic and cell differentiation processes. As such, our data provide a comprehensive view of understanding the transcriptional regulation mechanism during the differentiation of porcine subcutaneous preadipocytes.

Generally speaking, preadipocyte differentiation mainly consists of three important stages including growth arrest, mitotic clonal expansion, and late events and terminal differentiation [25–27]. In the early stage of differentiation (D2), we found that upregulated mRNAs were mainly enriched in lipid metabolic process, while downregulated mRNAs were closely involved in cell cycle-related processes (Fig. 4a and Additional file 8). Many key markers, e.g., *LIPE*, *APOE*, *PLIN1*, *DGAT2*, *ADIPOQ*, and *LPL* for lipid metabolism and *CDK1*, *CCND1*, and *E2F1* for cell cycle (Additional file 3), were identified in this study. Meanwhile, triglyceride contents at D2 were observed to increase but did not reach significant levels compared with those at D0, suggesting that upregulated expression levels of these lipid-related markers at the early stage of differentiation does not significantly alter triglyceride phenotype (Fig. 1b-c). However, further studies are needed to confirm this speculation. As expected, two critical well-documented markers involved in preadipocyte differentiation, *PPARG* and *CEBPA*, were shown to highly significant increase at D2 (Additional file 3). In the past few decades, *PPARG* and *CEBPA* have been deeply investigated for their determinant role in initiating and regulating preadipocyte differentiation [28, 29]. Besides, these two markers have been found to be involved in growth arrest of differentiation [30, 31]. Before preadipocyte differentiation, growth arrest is a necessary process for blocking the cell in the G_1_ phase [32, 33]; restricted cell proliferation has been found to appear at D2 of differentiation in human mesenchymal stem cells [32, 34]. In the G_1_/S phase, *CDK1*, *CCND1*, and *E2F1* have been shown to play a key role in regulating the cell cycle [35]. Furthermore, the expression of *CDK1* and *CCND1* decreased at the early stage of differentiation in human and porcine preadipocytes [34, 36]. Additionally, *PPARG* has been reported to control the cell cycle by decreasing the activity of E2F and CCND1 and upregulating the expression of cyclin-dependent kinase inhibitors [30, 37, 38], while *CEBPA* can repress the expression of *E2F1*, which results in the impairment of the ability of cell differentiation, and simultaneously suppresses cell proliferation [31]. Consistent with these data, our results found that *CDK1*, *CCND1*, and *E2F1* levels decreased significantly at D2, accompanying the increase in the levels of *PPARG* and *CEBPA*, demonstrating that *PPARG* and *CEBPA* might impact growth arrest at D2, via the downregulation of cell cycle-related markers.

Conversely, compared with the early stage of differentiation (D2), cell cycle-related marker levels were also found to increase at the middle stage of differentiation (D4) (Fig. 4b and Additional file 9). This is not surprising because preadipocytes reenter the cell cycle with at least one round of mitotic clonal expansion for increasing the proportion of adipocytes after the G_1_ phase growth-arrested; this is a synchronous process for adipogenesis [26]. Moreover, mitotic clonal expansion is mainly related to the composition of fat inducers, especially insulin [39]. Consistent with these results, our data supported that mitotic clonal expansion might be induced by insulin, which resulted in increased levels of cell cycle-related markers, e.g., *CDK1*, *CCNA2*, *CCNE2*, and *E2F1* (Additional file 4). As mentioned above, *CDK1* and *E2F1* are key markers for controlling the G_1_/S phase of the cell cycle. Furthermore, *CCNA2* is an important gene of the S phase of the cell cycle in combination with *CDK1*, while *CCNE2* is a critical factor of the G_1_/S phase of the cell cycle in combination with *E2F1* [35]. Hence, the upregulation of these genes might promote cell cycle process from D2 to D4. Interestingly, our data indicated that extracellular matrix-related genes such as *COL14A1* and *MFAP5* were downregulated at D4, compared to D2 (Additional files 4 and 9). *COL14A1* is a gene encoding fibril-associated collagen; it has been shown to have an antiproliferative role in reducing de novo DNA synthesis in 3T3-L1 preadipocytes [40]. *MFAP5* encodes a microfibril-associated glycoprotein and its expression levels were reduced during human preadipocyte differentiation [41]. Here, the downregulation of these extracellular matrix-related genes at D4 might be associated with the changes of the adipocyte morphology, but additional investigation is required to decipher the role of the extracellular matrix in porcine adipogenesis.

From D4 to D8, lipid droplets grow larger and eventually form the mature adipocytes. Interestingly, the levels of many important markers related to actin cytoskeleton remodeling, e.g., *ARPC2*, *ARPC3*, and *DSTN*, were found to significantly increase at the later stage of differentiation (Additional files 5 and 10). As two major components of the actin cytoskeleton, *ARPC2* and *ARPC3* play an important role in adherens junction and intracellular motility of lipid vesicles [42, 43]. The knockdown of the ARP2/3 complex severely disrupted adipocyte differentiation [44]. *DSTN* is another actin-depolymerizing factor, and its knockdown inhibited adipocyte differentiation of human stromal stem cells [45]. Because the actin cytoskeleton is closely related to lipid droplet formation of the adipocytes [46], upregulation of these actin cytoskeleton-related genes at D8 might contribute to the formation of mature adipocytes at the later stage of differentiation. Meanwhile, we observed that the levels of other well-investigated markers associated with lipid lipolysis, e.g., *ABHD5*, *LIPE*, *PNPLA2*, and *ACOX1*, were significantly reduced at D8, compared to D4 (Additional file 5). Previously, *ABHD5*, *LIPE*, and *PNPLA2* were confirmed to be the master regulators of tricylglycerol hydrolysis [47–49], while ACOX1 is the first enzyme involved in the fatty acid beta-oxidation process [50]. Combined with the increase in triglyceride levels, these data support the claim that the changes of triglyceride phenotype might result from the downregulation of lipid lipolysis-related markers.

More importantly, compared to the initial phase of differentiation (D0), we discovered that the common DE mRNAs during the entire differentiation process were mainly involved in lipid metabolic and cell differentiation processes (Additional file 14). The levels of many well-known key markers, e.g., *LIPE*, *PLIN1*, *DGAT2*, *PNPLA2*, *LPL*, and *SCD* for lipid metabolic process, *PPARG* for cell differentiation, and *APOE* and *ADIPOQ* for both these processes, changed significantly during the entire differentiation process (Additional file 13), suggesting that these markers might play critical roles in phenotypic changes during the differentiation. In addition, the co-expressed network of the common DE lncRNAs and their target common DE mRNAs revealed that 36 lncRNAs targeted 132 lipogenesis-related mRNAs, indicating that these lncRNAs might participate in adipogenesis by positively or negatively regulating their target mRNAs (Fig. 6 and Additional file 15). For example, the co-expressed network showed that multiple lncRNAs could interact with *PPARG*, which is a decisive marker in adipogenesis (Fig. 6 and Additional file 15). Consequently, our results provide new evidence that lncRNAs are involved in porcine preadipocyte differentiation.

## Conclusions

In summary, a genome-wide view of the expression profiling of mRNAs, lncRNAs, and circRNAs during porcine subcutaneous preadipocyte differentiation was investigated. Moreover, a large number of DE genes, which could contribute to the phenotypic changes in adipocytes at different stages of differentiation, were further identified. Our study provides a comprehensive basis for the expression levels of various RNAs during adipocyte differentiation, thus giving us a novel clue for understanding the mechanism of the molecular regulation of subcutaneous adipogenesis in pigs.

## Additional files

Additional file 1: Primers of quantitative real-time RT-PCR. (XLSX 71 kb)

Additional file 2: List of expressed genes during porcine preadipocyte differentiation. Gene ID, FPKM or SRPBM value, gene name, biotype, position and description of all acquired genes with FPKM or SRPBM ≥1 in more than 50% of samples during porcine preadipocyte differentiation. (XLSX 2094 kb)

Additional file 3: List of differentially expressed (DE) genes for D2 vs D0. Gene ID, fold change, gene name, biotype, and position of differentially expressed mRNAs, lncRNAs, and circRNAs between D2 versus D0. (XLSX 345 kb)

Additional file 4: List of differentially expressed (DE) genes for D4 vs D2. Gene ID, fold change, gene name, biotype, and position of differentially expressed mRNAs, lncRNAs, and circRNAs between D4 versus D2. (XLSX 167 kb)

Additional file 5: List of differentially expressed (DE) genes for D8 vs D4. Gene ID, fold change, gene name, biotype, and position of differentially expressed mRNAs, lncRNAs, and circRNAs between D8 versus D4. (XLSX 295 kb)

Additional file 6: List of differentially expressed (DE) genes for D4 vs D0. Gene ID, fold change, gene name, biotype, and position of differentially expressed mRNAs, lncRNAs, and circRNAs between D4 versus D0. (XLSX 385 kb)

Additional file 7: List of differentially expressed (DE) genes for D8 vs D0. Gene ID, fold change, gene name, biotype, and position of differentially expressed mRNAs, lncRNAs, and circRNAs between D8 versus D0. (XLSX 206 kb)

Additional file 8: GO terms for upregulated and downregulated mRNAs, lncRNAs, and circRNAs from D2 vs D0. Terms marked gray background represent significant enrichment, while other terms represent non-significant enrichment. (XLSX 4497 kb)

Additional file 9: GO terms for upregulated and downregulated mRNAs, lncRNAs, and circRNAs from D4 vs D2. Terms marked gray background represent significant enrichment, while other terms represent non-significant enrichment. (XLSX 3928 kb)

Additional file 10: GO terms for upregulated and downregulated mRNAs, lncRNAs, and circRNAs from D8 vs D4. Terms marked gray background represent significant enrichment, while other terms represent non-significant enrichment. (XLSX 4067 kb)

Additional file 11: GO terms for upregulated and downregulated mRNAs, lncRNAs, and circRNAs from D4 vs D0. Terms marked gray background represent significant enrichment, while other terms represent non-significant enrichment. (XLSX 4373 kb)

Additional file 12: GO terms for upregulated and downregulated mRNAs, lncRNAs, and circRNAs from D8 vs D0. Terms marked gray background represent significant enrichment, while other terms represent non-significant enrichment. (XLSX 3796 kb)

Additional file 13: List of common differentially expressed (DE) genes for three differentiation stages (D2, D4, and D8) vs D0. Gene ID, fold change, gene name, biotype, and position of common differentially expressed (DE) mRNAs, lncRNAs, and circRNAs. (XLSX 5248 kb)

Additional file 14: GO terms for common differentially expressed (DE) mRNAs, lncRNAs, and circRNAs from three differentiation stages (D2, D4, and D8) versus D0. Terms marked gray background represent significant enrichment, while other terms represent non-significant enrichment. (XLSX 1763 kb)

Additional file 15: The relationship of common DE lncRNAs and their targeted common DE mRNAs involved in lipid metabolic and cell differentiation processes. (XLSX 34 kb)

## Abbreviations

ANOVA: Analysis of variance
BWA-MEM: Burrows-Wheeler Aligner-maximal exact match
CircRNA: Circular RNA
CIRI: CircRNA identifier
CNCI: Coding-non-coding index
CPAT: Coding potential assessing tool
CPC: Coding potential calculator
D0: Day 0
D2: Day 2
D4: Day 4
D8: Day 8
DE: Differentially expressed
DMI: Dexamethasone, 3-isobutyl-1-methylxanthine, and insulin
FBS: Fetal bovine serum
FDR: False discovery rate
FPKM: Fragments per kilobase of transcript per million reads mapped
GEO: Gene expression omnibus
GO: Gene ontology
IMF: Intramuscular fat
kb: kilobase pairs
LncRNA: Long non-coding RNA
MiRNA: MicroRNA
mRNA: Messenger RNA
PBS: Phosphate-buffered saline
PFAM: Protein folding domain database
qRT-PCR: Quantitative real-time RT-PCR
RIN: RNA integrity number
RNA-Seq: RNA sequencing
rRNA: Ribosomal RNA
SnoRNA: Small nucleolar RNA
SnRNA: Small nuclear RNA
SRPBM: Spliced reads per billion mapping
tRNA: Transfer RNA
